# *catena*-Poly[[[(dimethyl­malonato-κ^2^ *O*:*O*′)(perchlorato-κ*O*)copper(II)]-μ-bis­(3-pyridylmeth­yl)piperazinediium-κ^2^ *N* ^1′^:*N* ^4′^] perchlorate dihydrate]

**DOI:** 10.1107/S1600536808036490

**Published:** 2008-11-13

**Authors:** Gregory A. Farnum, Robert L. LaDuca

**Affiliations:** a Lyman Briggs College, Department of Chemistry, Michigan State University, East Lansing, MI 48825 USA

## Abstract

In the title compound, {[Cu(C_5_H_6_O_4_)(ClO_4_)(C_16_H_22_N_4_)]ClO_4_·2H_2_O}_*n*_, square-pyramidally coordinated Cu atoms with perchlorate and dimethyl­malonate ligands are connected into cationic sinusoidal coordination polymer chains by doubly protonated bis­(3-pyridylmeth­yl)piperazine (3-bpmp) ligands. The chains aggregate into pseudo-layers parallel to the (101) crystal planes by N—H⋯O hydrogen bonding. Unligated perchlorate anions and water mol­ecules of crystallization provide additional hydrogen bonding between pseudo-layers.

## Related literature

For copper carboxyl­ate coordination polymers containing 3-bpmp, see: Johnston *et al.* (2008 ▶). For the synthesis of 3-bpmp, see: Pocic *et al.* (2005 ▶).
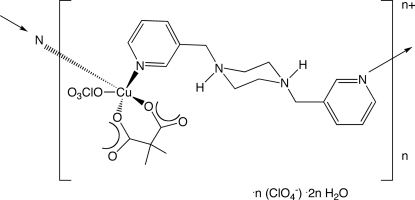


## Experimental

### 

#### Crystal data

[Cu(C_5_H_6_O_4_)(ClO_4_)(C_16_H_22_N_4_)]ClO_4_·2H_2_O
*M* *_r_* = 698.95
Triclinic, 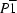

*a* = 9.6284 (15) Å
*b* = 10.5140 (16) Å
*c* = 14.061 (2) Å
α = 86.950 (2)°
β = 82.634 (2)°
γ = 84.638 (2)°
*V* = 1404.3 (4) Å^3^
*Z* = 2
Mo *K*α radiation
μ = 1.04 mm^−1^
*T* = 173 (2) K
0.40 × 0.30 × 0.15 mm

#### Data collection

Bruker SMART 1K diffractometer
Absorption correction: multi-scan (*SADABS*; Sheldrick, 1996 ▶) *T*_min_ = 0.680, *T*_max_ = 0.859
14378 measured reflections
6309 independent reflections
4904 reflections with *I* > 2σ(*I*)
*R*_int_ = 0.035

#### Refinement

*R*[*F* ^2^ > 2σ(*F* ^2^)] = 0.040
*wR*(*F* ^2^) = 0.099
*S* = 1.05
6309 reflections
397 parameters
8 restraints
H atoms treated by a mixture of independent and constrained refinement
Δρ_max_ = 0.57 e Å^−3^
Δρ_min_ = −0.44 e Å^−3^

### 

Data collection: *SMART* (Bruker, 2007 ▶); cell refinement: *SAINT* (Bruker, 2007 ▶); data reduction: *SAINT*; program(s) used to solve structure: *SHELXS97* (Sheldrick, 2008 ▶); program(s) used to refine structure: *SHELXL97* (Sheldrick, 2008 ▶); molecular graphics: *Crystal Maker* (Palmer, 2007 ▶); software used to prepare material for publication: *SHELXL97*.

## Supplementary Material

Crystal structure: contains datablocks I, global. DOI: 10.1107/S1600536808036490/lh2730sup1.cif

Structure factors: contains datablocks I. DOI: 10.1107/S1600536808036490/lh2730Isup2.hkl

Additional supplementary materials:  crystallographic information; 3D view; checkCIF report

## Figures and Tables

**Table 1:** Hydrogen-bond geometry (Å, °)

*D*—H⋯*A*	*D*—H	H⋯*A*	*D*⋯*A*	*D*—H⋯*A*
O1*W*—H1*WA*⋯O12	0.894 (18)	1.98 (2)	2.838 (4)	160 (4)
O1*W*—H1*WB*⋯O8^i^	0.875 (18)	2.35 (3)	3.053 (4)	137 (3)
O1*W*—H1*WB*⋯O2^ii^	0.875 (18)	2.46 (3)	3.120 (3)	133 (3)
O2*W*—H2*WA*⋯O7	0.929 (19)	2.14 (2)	3.044 (4)	164 (4)
O2*W*—H2*WB*⋯O1*W*^iii^	0.941 (19)	1.95 (3)	2.807 (5)	150 (5)
N2—H2*N*⋯O2^iv^	0.887 (17)	1.804 (18)	2.673 (3)	166 (3)
N4—H4*N*⋯O4^v^	0.923 (17)	1.727 (17)	2.647 (3)	175 (3)

Symmetry codes: (i) 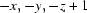; (ii) 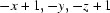; (iii) 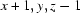; (iv) 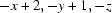; (v) 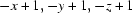.

**Fig. 1.**
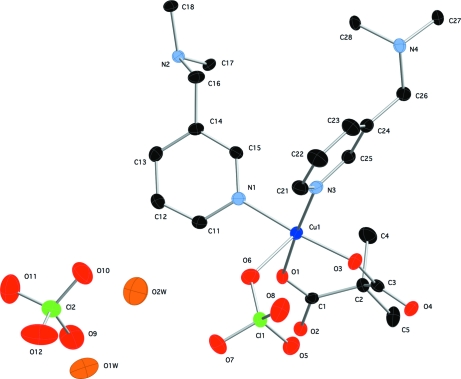
Coordination environment of the title compound, showing 50% probability ellipsoids and atom numbering scheme. Hydrogen atoms are not shown. Color codes: dark blue Cu, light blue N, red O, black C, green Cl and orange O atoms of water molecules.

**Fig. 2.**
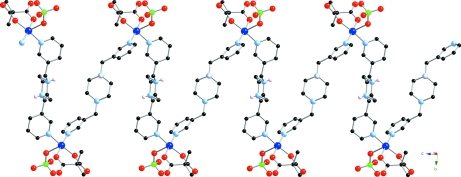
A single cationic [Cu(dimethylmalonate)(ClO4)(3-bpmpH2)]n chain in the title compound.

**Fig. 3.**
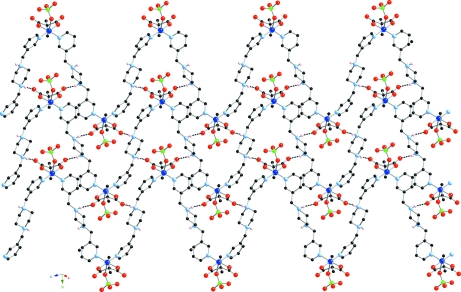
A supramolecular [Cu(dimethylmalonate)(ClO4)(3-bpmpH2)]n layer in the title compound. Hydrogen bonding between protonated piperazinyl N atoms and unligated dimethylmalonate carboxylate O atoms is shown as dashed lines.

**Fig. 4.**
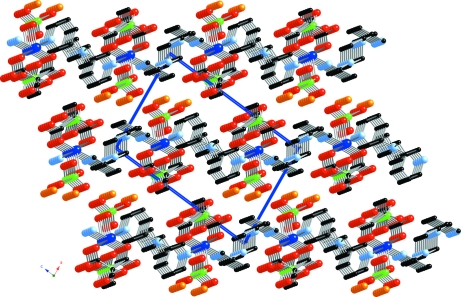
Packing diagram illustrating the interaction of pseudolayers via hydrogen bonding involving the dimethylmalonate carboxylate O atoms, co-crystallized water molecules, and unligated perchlorate anions.

## supplementary crystallographic information

### Comment

In comparison to coordination polymers based on the rigid rod tether 4,4'- bipyridine, extended phases based on the flexible and hydrogen-bonding capable bis(3-pyridylmethyl)piperazine (3-bpmp) ligand are much less common (Johnston *et al.*, 2008).

The asymmetric unit of the title compound contains a divalent copper atom, two halves of two 3-bpmp molecules protonated at their piperazinyl nitrogen atoms, one dimethylmalonate dianion, one bound and one unbound perchlorate ion and two water molecules of crystallization (Figure 1). The Cu atoms are square pyramidally coordinated in a {CuN_2_O_3_} arrangement, with the basal plane occupied by two *cis* N atom donors from two crystallographically distinct 3-bpmp ligands and two *cis* O atom donors from a dimethylmalonate ligand in a 1,3-chelating binding mode. The apical position is filled by a ligated perchlorate anion.

The 3-bpmp ligands link the Cu atoms into sinusoidal cationic coordination polymer chains with formulation [Cu(3-bpmpH_2_)(dimethylmalonate)(ClO_4_)]_n_^n+^ (Figure 2), in which the through ligand Cu···Cu contact distance is 15.441 Å. The "wavelength" of this chain, defined by unbridged Cu···Cu contacts, is 15.991 Å. The chains are aligned parallel to the [1 0 1] crystal direction.

Neighboring chains interdigitate and aggregate into a pseudolayer (Figure 3) parallel to the (1 0 1) crystal plane by N—H···O hydrogen bonding between protonated piperazinyl N atoms and unligated dimethylmalonate O atoms. These stack into three dimensions (Figure 4) through additional hydrogen bonding patterns involving unligated perchlorate anions and water molecule dimers, and dimethylmalonate O atoms within the coordination polymer chains.

### Experimental

All chemicals were obtained commercially, except for 3-bpmp which was synthesized by a literature method (Pocic *et al.*, 2005). Copper perchlorate hexahydrate (19 mg, 0.051 mmol) and dimethylmalonic acid (7 mg, 0.05 mmol) were dissolved in 3 ml water in a glass vial. A 1 ml aliquot of a 1:1 water:ethanol solution was carefully layered onto the aqueous solution, followed by 3 ml of an ethanolic solution of 3-bpmp (27 mg, 0.10 mmol). Blue blocks of the title compound formed after 1 week.

### Refinement

All H atoms bound to C atoms were placed in calculated positions, with C—H = 0.95 - 0.99 Å and refined in riding mode with *U*_iso_ = 1.2*U*_eq_(C) or 1.5U_eq_(C) for methyl C atoms. All H atoms bound to O atoms were found *via* Fourier difference map, restrained with O—H = 0.89 Å, and refined with *U*_iso_=1.2*U*_eq_(O). All H atoms bound to N atoms were found *via* Fourier difference map, restrained with N—H = 0.89 Å, and refined with *U*_iso_=1.2*U*_eq_(N).

### Crystal data

**Table:**

[Cu(C_5_H_6_O_4_)(ClO_4_)(C_16_H_22_N_4_)]ClO_4_·2H_2_O	*Z* = 2
*M**_r_* = 698.95	*F*_000_ = 722
Triclinic, *P*1	*D*_x_ = 1.653 Mg m^−^^3^
*a* = 9.6284 (15) Å	Mo *K*α radiation λ = 0.71073 Å
*b* = 10.5140 (16) Å	Cell parameters from 14378 reflections
*c* = 14.061 (2) Å	θ = 1.5–28.3º
α = 86.950 (2)º	µ = 1.04 mm^−^^1^
β = 82.634 (2)º	*T* = 173 (2) K
γ = 84.638 (2)º	Block, blue
*V* = 1404.3 (4) Å^3^	0.40 × 0.30 × 0.15 mm

### Data collection

**Table:**

Bruker SMART 1K diffractometer	6309 independent reflections
Radiation source: fine-focus sealed tube	4904 reflections with *I* > 2σ(*I*)
Monochromator: graphite	*R*_int_ = 0.035
*T* = 173(2) K	θ_max_ = 28.3º
ω scans	θ_min_ = 1.5º
Absorption correction: multi-scan(SADABS; Sheldrick, 1996)	*h* = −12→12
*T*_min_ = 0.680, *T*_max_ = 0.859	*k* = −13→13
14378 measured reflections	*l* = −18→18

### Refinement

**Table:**

Refinement on *F*^2^	Secondary atom site location: difference Fourier map
Least-squares matrix: full	Hydrogen site location: inferred from neighbouring sites
*R*[*F*^2^ > 2σ(*F*^2^)] = 0.040	H atoms treated by a mixture of independent and constrained refinement
*wR*(*F*^2^) = 0.099	*w* = 1/[σ^2^(*F*_o_^2^) + (0.0402*P*)^2^ + 1.0163*P*] where *P* = (*F*_o_^2^ + 2*F*_c_^2^)/3
*S* = 1.05	(Δ/σ)_max_ < 0.001
6309 reflections	Δρ_max_ = 0.57 e Å^−^^3^
397 parameters	Δρ_min_ = −0.44 e Å^−^^3^
8 restraints	Extinction correction: none
Primary atom site location: structure-invariant direct methods	

### Special details

**Table:**

Geometry. All e.s.d.'s (except the e.s.d. in the dihedral angle between two l.s. planes) are estimated using the full covariance matrix. The cell e.s.d.'s are taken into account individually in the estimation of e.s.d.'s in distances, angles and torsion angles; correlations between e.s.d.'s in cell parameters are only used when they are defined by crystal symmetry. An approximate (isotropic) treatment of cell e.s.d.'s is used for estimating e.s.d.'s involving l.s. planes.
Refinement. Refinement of *F*^2^ against ALL reflections. The weighted *R*-factor *wR* and goodness of fit *S* are based on *F*^2^, conventional *R*-factors *R* are based on *F*, with *F* set to zero for negative *F*^2^. The threshold expression of *F*^2^ > σ(*F*^2^) is used only for calculating *R*-factors(gt) *etc*. and is not relevant to the choice of reflections for refinement. *R*-factors based on *F*^2^ are statistically about twice as large as those based on *F*, and *R*- factors based on ALL data will be even larger.

### Fractional atomic coordinates and isotropic or equivalent isotropic displacement parameters (Å^2^)

**Table:**

	*x*	*y*	*z*	*U*_iso_*/*U*_eq_	
Cu1	0.73682 (3)	0.38286 (3)	0.22768 (2)	0.01649 (9)	
Cl1	0.52659 (6)	0.15993 (6)	0.15616 (4)	0.01940 (14)	
Cl2	0.04531 (7)	0.26708 (7)	0.67794 (5)	0.02767 (17)	
O1	0.89350 (18)	0.26684 (16)	0.17600 (12)	0.0185 (4)	
O1W	−0.3036 (3)	0.0931 (2)	0.7673 (2)	0.0524 (7)	
H1WA	−0.221 (3)	0.124 (3)	0.747 (3)	0.063*	
H1WB	−0.284 (4)	0.013 (2)	0.785 (3)	0.063*	
O2	1.06366 (18)	0.11347 (16)	0.18831 (12)	0.0196 (4)	
O2W	0.6049 (5)	0.2872 (3)	−0.1037 (2)	0.0946 (12)	
H2WA	0.573 (6)	0.247 (4)	−0.045 (2)	0.114*	
H2WB	0.602 (6)	0.218 (4)	−0.143 (3)	0.114*	
O3	0.71336 (18)	0.28139 (17)	0.34603 (13)	0.0211 (4)	
O4	0.78008 (19)	0.12856 (17)	0.44779 (13)	0.0211 (4)	
O5	0.6183 (2)	0.07458 (18)	0.20754 (14)	0.0282 (5)	
O6	0.5848 (2)	0.28179 (18)	0.13822 (15)	0.0287 (5)	
O7	0.5127 (2)	0.1087 (2)	0.06554 (15)	0.0362 (5)	
O8	0.3908 (2)	0.1798 (2)	0.21162 (16)	0.0370 (5)	
O9	0.1580 (3)	0.1796 (2)	0.70439 (19)	0.0536 (7)	
O10	0.0031 (3)	0.3535 (2)	0.75267 (17)	0.0514 (7)	
O11	0.0911 (3)	0.3375 (3)	0.59195 (18)	0.0571 (7)	
O12	−0.0708 (3)	0.2017 (2)	0.6593 (2)	0.0650 (9)	
N1	0.7670 (2)	0.50054 (19)	0.11139 (15)	0.0167 (5)	
N2	0.8888 (2)	0.91571 (19)	0.00109 (15)	0.0144 (4)	
H2N	0.908 (3)	0.893 (2)	−0.0593 (13)	0.017*	
N3	0.5816 (2)	0.50287 (19)	0.29083 (15)	0.0182 (5)	
N4	0.4942 (2)	0.8624 (2)	0.49802 (15)	0.0156 (4)	
H4N	0.3982 (18)	0.863 (3)	0.514 (2)	0.019*	
C1	0.9729 (3)	0.1943 (2)	0.22604 (18)	0.0153 (5)	
C2	0.9626 (3)	0.2108 (2)	0.33488 (18)	0.0166 (5)	
C3	0.8085 (3)	0.2046 (2)	0.37928 (18)	0.0161 (5)	
C4	1.0062 (3)	0.3455 (3)	0.3502 (2)	0.0300 (7)	
H4A	0.9451	0.4102	0.3190	0.045*	
H4B	1.1041	0.3516	0.3223	0.045*	
H4C	0.9969	0.3600	0.4191	0.045*	
C5	1.0578 (3)	0.1103 (3)	0.3830 (2)	0.0255 (6)	
H5A	1.0483	0.1242	0.4520	0.038*	
H5B	1.1557	0.1172	0.3553	0.038*	
H5C	1.0309	0.0250	0.3728	0.038*	
C11	0.7887 (3)	0.4505 (3)	0.02408 (19)	0.0221 (6)	
H11	0.7937	0.3602	0.0201	0.027*	
C12	0.8040 (3)	0.5242 (3)	−0.0596 (2)	0.0246 (6)	
H12	0.8197	0.4854	−0.1200	0.029*	
C13	0.7962 (3)	0.6561 (3)	−0.05452 (19)	0.0211 (6)	
H13	0.8042	0.7092	−0.1114	0.025*	
C14	0.7766 (3)	0.7096 (2)	0.03496 (19)	0.0169 (5)	
C15	0.7619 (3)	0.6285 (2)	0.11544 (19)	0.0166 (5)	
H15	0.7475	0.6649	0.1767	0.020*	
C16	0.7611 (3)	0.8529 (2)	0.0465 (2)	0.0196 (6)	
H16A	0.6780	0.8906	0.0169	0.024*	
H16B	0.7447	0.8704	0.1157	0.024*	
C17	1.0141 (3)	0.8795 (2)	0.05230 (18)	0.0164 (5)	
H17A	1.0344	0.7855	0.0539	0.020*	
H17B	0.9939	0.9070	0.1193	0.020*	
C18	0.8594 (3)	1.0583 (2)	−0.00249 (19)	0.0165 (5)	
H18A	0.8365	1.0888	0.0636	0.020*	
H18B	0.7774	1.0827	−0.0375	0.020*	
C21	0.4487 (3)	0.5093 (3)	0.2703 (2)	0.0247 (6)	
H21	0.4271	0.4612	0.2197	0.030*	
C22	0.3425 (3)	0.5851 (3)	0.3216 (2)	0.0307 (7)	
H22	0.2487	0.5871	0.3069	0.037*	
C23	0.3727 (3)	0.6571 (3)	0.3935 (2)	0.0254 (6)	
H23	0.3001	0.7085	0.4293	0.031*	
C24	0.5109 (3)	0.6542 (2)	0.41357 (18)	0.0187 (5)	
C25	0.6108 (3)	0.5742 (2)	0.36102 (18)	0.0176 (5)	
H25	0.7050	0.5694	0.3753	0.021*	
C26	0.5575 (3)	0.7270 (2)	0.49164 (19)	0.0196 (6)	
H26A	0.5329	0.6812	0.5540	0.023*	
H26B	0.6611	0.7272	0.4807	0.023*	
C27	0.5498 (3)	0.9241 (2)	0.57676 (18)	0.0194 (6)	
H27A	0.6525	0.9282	0.5606	0.023*	
H27B	0.5336	0.8714	0.6369	0.023*	
C28	0.5198 (3)	0.9422 (2)	0.40762 (18)	0.0174 (5)	
H28A	0.4809	0.9038	0.3551	0.021*	
H28B	0.6222	0.9457	0.3891	0.021*	

### Atomic displacement parameters (Å^2^)

**Table:**

	*U*^11^	*U*^22^	*U*^33^	*U*^12^	*U*^13^	*U*^23^
Cu1	0.01812 (17)	0.01517 (16)	0.01464 (17)	0.00267 (12)	0.00069 (12)	−0.00020 (12)
Cl1	0.0194 (3)	0.0213 (3)	0.0175 (3)	−0.0024 (2)	−0.0016 (2)	−0.0016 (2)
Cl2	0.0217 (3)	0.0271 (4)	0.0340 (4)	−0.0028 (3)	−0.0008 (3)	−0.0043 (3)
O1	0.0208 (9)	0.0205 (9)	0.0129 (9)	0.0050 (7)	−0.0027 (7)	−0.0007 (7)
O1W	0.0443 (15)	0.0369 (14)	0.074 (2)	−0.0042 (12)	−0.0019 (14)	0.0062 (14)
O2	0.0199 (9)	0.0211 (9)	0.0160 (9)	0.0040 (8)	0.0009 (7)	−0.0013 (7)
O2W	0.160 (4)	0.070 (2)	0.050 (2)	−0.014 (2)	0.005 (2)	0.0045 (17)
O3	0.0181 (9)	0.0240 (10)	0.0182 (10)	0.0053 (8)	0.0020 (7)	0.0040 (8)
O4	0.0197 (9)	0.0232 (10)	0.0179 (10)	0.0010 (8)	0.0024 (7)	0.0048 (8)
O5	0.0284 (11)	0.0276 (11)	0.0268 (11)	0.0047 (9)	−0.0029 (9)	0.0028 (9)
O6	0.0333 (11)	0.0223 (10)	0.0330 (12)	−0.0087 (9)	−0.0098 (9)	0.0009 (9)
O7	0.0451 (13)	0.0387 (13)	0.0287 (12)	−0.0031 (10)	−0.0144 (10)	−0.0129 (10)
O8	0.0193 (10)	0.0534 (14)	0.0336 (12)	0.0031 (10)	0.0056 (9)	0.0099 (11)
O9	0.0453 (15)	0.0555 (16)	0.0568 (17)	0.0255 (12)	−0.0116 (13)	−0.0118 (13)
O10	0.0655 (17)	0.0493 (15)	0.0382 (14)	0.0187 (13)	−0.0128 (13)	−0.0165 (12)
O11	0.0573 (17)	0.079 (2)	0.0366 (15)	−0.0206 (15)	−0.0037 (12)	0.0089 (14)
O12	0.0332 (14)	0.0416 (15)	0.123 (3)	−0.0146 (11)	−0.0043 (15)	−0.0191 (16)
N1	0.0172 (11)	0.0154 (10)	0.0169 (11)	−0.0011 (8)	0.0000 (9)	−0.0012 (9)
N2	0.0158 (10)	0.0127 (10)	0.0138 (11)	−0.0002 (8)	0.0014 (8)	−0.0008 (8)
N3	0.0207 (11)	0.0150 (10)	0.0175 (12)	0.0014 (9)	0.0005 (9)	0.0010 (9)
N4	0.0148 (10)	0.0183 (11)	0.0130 (11)	−0.0004 (9)	0.0012 (8)	−0.0016 (8)
C1	0.0150 (12)	0.0135 (12)	0.0169 (13)	−0.0041 (10)	0.0018 (10)	0.0004 (10)
C2	0.0165 (13)	0.0186 (13)	0.0148 (13)	−0.0019 (10)	−0.0015 (10)	−0.0015 (10)
C3	0.0177 (13)	0.0142 (12)	0.0158 (13)	0.0000 (10)	0.0013 (10)	−0.0052 (10)
C4	0.0348 (17)	0.0286 (16)	0.0288 (17)	−0.0155 (13)	−0.0019 (13)	−0.0060 (13)
C5	0.0197 (14)	0.0377 (17)	0.0172 (14)	0.0049 (12)	−0.0015 (11)	0.0023 (12)
C11	0.0282 (15)	0.0166 (13)	0.0208 (14)	0.0000 (11)	−0.0007 (11)	−0.0025 (11)
C12	0.0336 (16)	0.0228 (14)	0.0167 (14)	−0.0034 (12)	0.0009 (12)	−0.0040 (11)
C13	0.0248 (14)	0.0204 (13)	0.0175 (14)	−0.0060 (11)	0.0005 (11)	0.0030 (11)
C14	0.0139 (12)	0.0141 (12)	0.0224 (14)	−0.0037 (10)	0.0003 (10)	0.0005 (10)
C15	0.0174 (13)	0.0161 (12)	0.0164 (13)	−0.0031 (10)	−0.0006 (10)	−0.0028 (10)
C16	0.0171 (13)	0.0155 (12)	0.0250 (15)	−0.0033 (10)	0.0037 (11)	0.0000 (11)
C17	0.0189 (13)	0.0128 (12)	0.0170 (13)	0.0008 (10)	−0.0031 (10)	0.0029 (10)
C18	0.0165 (12)	0.0117 (11)	0.0203 (14)	0.0010 (10)	−0.0009 (10)	0.0003 (10)
C21	0.0272 (15)	0.0213 (14)	0.0261 (15)	0.0033 (12)	−0.0073 (12)	−0.0052 (12)
C22	0.0218 (15)	0.0313 (16)	0.0394 (19)	0.0047 (12)	−0.0073 (13)	−0.0101 (14)
C23	0.0203 (14)	0.0266 (15)	0.0283 (16)	0.0031 (12)	0.0004 (12)	−0.0088 (12)
C24	0.0209 (13)	0.0172 (13)	0.0172 (13)	−0.0013 (10)	0.0000 (11)	0.0011 (10)
C25	0.0177 (13)	0.0171 (12)	0.0175 (13)	−0.0008 (10)	−0.0007 (10)	0.0004 (10)
C26	0.0198 (13)	0.0186 (13)	0.0190 (14)	0.0035 (11)	−0.0010 (11)	−0.0020 (11)
C27	0.0244 (14)	0.0211 (13)	0.0133 (13)	−0.0012 (11)	−0.0041 (11)	−0.0018 (10)
C28	0.0212 (13)	0.0197 (13)	0.0107 (12)	−0.0030 (10)	0.0016 (10)	−0.0018 (10)

### Geometric parameters (Å, °)

**Table:**

Cu1—O3	1.9283 (18)	C4—H4C	0.9800
Cu1—O1	1.9394 (17)	C5—H5A	0.9800
Cu1—N1	2.005 (2)	C5—H5B	0.9800
Cu1—N3	2.010 (2)	C5—H5C	0.9800
Cu1—O6	2.400 (2)	C11—C12	1.374 (4)
Cl1—O5	1.433 (2)	C11—H11	0.9500
Cl1—O7	1.436 (2)	C12—C13	1.387 (4)
Cl1—O8	1.438 (2)	C12—H12	0.9500
Cl1—O6	1.443 (2)	C13—C14	1.389 (4)
Cl2—O10	1.420 (2)	C13—H13	0.9500
Cl2—O12	1.425 (3)	C14—C15	1.381 (3)
Cl2—O11	1.426 (3)	C14—C16	1.516 (3)
Cl2—O9	1.429 (2)	C15—H15	0.9500
O1—C1	1.278 (3)	C16—H16A	0.9900
O1W—H1WA	0.894 (18)	C16—H16B	0.9900
O1W—H1WB	0.875 (18)	C17—C18^i^	1.511 (3)
O2—C1	1.248 (3)	C17—H17A	0.9900
O2W—H2WA	0.929 (19)	C17—H17B	0.9900
O2W—H2WB	0.941 (19)	C18—C17^i^	1.511 (3)
O3—C3	1.281 (3)	C18—H18A	0.9900
O4—C3	1.239 (3)	C18—H18B	0.9900
N1—C11	1.345 (3)	C21—C22	1.386 (4)
N1—C15	1.346 (3)	C21—H21	0.9500
N2—C17	1.492 (3)	C22—C23	1.370 (4)
N2—C18	1.499 (3)	C22—H22	0.9500
N2—C16	1.503 (3)	C23—C24	1.392 (4)
N2—H2N	0.887 (17)	C23—H23	0.9500
N3—C21	1.342 (4)	C24—C25	1.383 (3)
N3—C25	1.343 (3)	C24—C26	1.507 (4)
N4—C28	1.492 (3)	C25—H25	0.9500
N4—C27	1.493 (3)	C26—H26A	0.9900
N4—C26	1.498 (3)	C26—H26B	0.9900
N4—H4N	0.923 (17)	C27—C28^ii^	1.514 (3)
C1—C2	1.539 (4)	C27—H27A	0.9900
C2—C5	1.522 (3)	C27—H27B	0.9900
C2—C3	1.540 (3)	C28—C27^ii^	1.514 (3)
C2—C4	1.548 (4)	C28—H28A	0.9900
C4—H4A	0.9800	C28—H28B	0.9900
C4—H4B	0.9800		
			
O3—Cu1—O1	91.49 (7)	H5B—C5—H5C	109.5
O3—Cu1—N1	174.84 (8)	N1—C11—C12	122.9 (2)
O1—Cu1—N1	90.38 (8)	N1—C11—H11	118.6
O3—Cu1—N3	85.44 (8)	C12—C11—H11	118.6
O1—Cu1—N3	175.47 (8)	C11—C12—C13	118.9 (3)
N1—Cu1—N3	92.41 (8)	C11—C12—H12	120.5
O3—Cu1—O6	99.44 (7)	C13—C12—H12	120.5
O1—Cu1—O6	89.56 (7)	C12—C13—C14	119.1 (2)
N1—Cu1—O6	85.38 (8)	C12—C13—H13	120.5
N3—Cu1—O6	94.23 (8)	C14—C13—H13	120.5
O5—Cl1—O7	110.03 (13)	C15—C14—C13	118.2 (2)
O5—Cl1—O8	110.26 (12)	C15—C14—C16	119.5 (2)
O7—Cl1—O8	109.95 (14)	C13—C14—C16	122.2 (2)
O5—Cl1—O6	109.60 (12)	N1—C15—C14	123.3 (2)
O7—Cl1—O6	108.42 (13)	N1—C15—H15	118.4
O8—Cl1—O6	108.54 (13)	C14—C15—H15	118.4
O10—Cl2—O12	110.28 (17)	N2—C16—C14	112.3 (2)
O10—Cl2—O11	109.08 (17)	N2—C16—H16A	109.1
O12—Cl2—O11	107.14 (19)	C14—C16—H16A	109.1
O10—Cl2—O9	109.14 (15)	N2—C16—H16B	109.1
O12—Cl2—O9	111.41 (17)	C14—C16—H16B	109.1
O11—Cl2—O9	109.75 (16)	H16A—C16—H16B	107.9
C1—O1—Cu1	125.13 (16)	N2—C17—C18^i^	110.5 (2)
H1WA—O1W—H1WB	106 (3)	N2—C17—H17A	109.5
H2WA—O2W—H2WB	98 (3)	C18^i^—C17—H17A	109.5
C3—O3—Cu1	125.71 (16)	N2—C17—H17B	109.5
Cl1—O6—Cu1	129.66 (12)	C18^i^—C17—H17B	109.5
C11—N1—C15	117.6 (2)	H17A—C17—H17B	108.1
C11—N1—Cu1	118.88 (17)	N2—C18—C17^i^	110.23 (19)
C15—N1—Cu1	123.45 (17)	N2—C18—H18A	109.6
C17—N2—C18	109.43 (19)	C17^i^—C18—H18A	109.6
C17—N2—C16	112.43 (19)	N2—C18—H18B	109.6
C18—N2—C16	110.56 (18)	C17^i^—C18—H18B	109.6
C17—N2—H2N	109.4 (18)	H18A—C18—H18B	108.1
C18—N2—H2N	106.6 (17)	N3—C21—C22	121.2 (3)
C16—N2—H2N	108.2 (18)	N3—C21—H21	119.4
C21—N3—C25	118.6 (2)	C22—C21—H21	119.4
C21—N3—Cu1	123.14 (19)	C23—C22—C21	120.0 (3)
C25—N3—Cu1	118.18 (17)	C23—C22—H22	120.0
C28—N4—C27	108.84 (19)	C21—C22—H22	120.0
C28—N4—C26	114.45 (19)	C22—C23—C24	119.3 (3)
C27—N4—C26	109.3 (2)	C22—C23—H23	120.3
C28—N4—H4N	107.8 (17)	C24—C23—H23	120.3
C27—N4—H4N	107.2 (18)	C25—C24—C23	117.4 (2)
C26—N4—H4N	109.1 (17)	C25—C24—C26	117.9 (2)
O2—C1—O1	121.6 (2)	C23—C24—C26	124.5 (2)
O2—C1—C2	118.6 (2)	N3—C25—C24	123.4 (2)
O1—C1—C2	119.7 (2)	N3—C25—H25	118.3
C5—C2—C1	112.0 (2)	C24—C25—H25	118.3
C5—C2—C3	110.5 (2)	N4—C26—C24	114.5 (2)
C1—C2—C3	108.9 (2)	N4—C26—H26A	108.6
C5—C2—C4	109.5 (2)	C24—C26—H26A	108.6
C1—C2—C4	107.7 (2)	N4—C26—H26B	108.6
C3—C2—C4	108.1 (2)	C24—C26—H26B	108.6
O4—C3—O3	121.7 (2)	H26A—C26—H26B	107.6
O4—C3—C2	119.4 (2)	N4—C27—C28^ii^	111.7 (2)
O3—C3—C2	118.9 (2)	N4—C27—H27A	109.3
C2—C4—H4A	109.5	C28^ii^—C27—H27A	109.3
C2—C4—H4B	109.5	N4—C27—H27B	109.3
H4A—C4—H4B	109.5	C28^ii^—C27—H27B	109.3
C2—C4—H4C	109.5	H27A—C27—H27B	107.9
H4A—C4—H4C	109.5	N4—C28—C27^ii^	109.3 (2)
H4B—C4—H4C	109.5	N4—C28—H28A	109.8
C2—C5—H5A	109.5	C27^ii^—C28—H28A	109.8
C2—C5—H5B	109.5	N4—C28—H28B	109.8
H5A—C5—H5B	109.5	C27^ii^—C28—H28B	109.8
C2—C5—H5C	109.5	H28A—C28—H28B	108.3
H5A—C5—H5C	109.5		
			
O3—Cu1—O1—C1	−25.1 (2)	C1—C2—C3—O3	−55.3 (3)
N1—Cu1—O1—C1	150.0 (2)	C4—C2—C3—O3	61.4 (3)
O6—Cu1—O1—C1	−124.6 (2)	C15—N1—C11—C12	−0.7 (4)
O1—Cu1—O3—C3	23.3 (2)	Cu1—N1—C11—C12	177.0 (2)
N3—Cu1—O3—C3	−153.4 (2)	N1—C11—C12—C13	−0.3 (4)
O6—Cu1—O3—C3	113.1 (2)	C11—C12—C13—C14	1.5 (4)
O5—Cl1—O6—Cu1	−27.77 (19)	C12—C13—C14—C15	−1.7 (4)
O7—Cl1—O6—Cu1	−147.89 (15)	C12—C13—C14—C16	−177.5 (2)
O8—Cl1—O6—Cu1	92.71 (17)	C11—N1—C15—C14	0.5 (4)
O3—Cu1—O6—Cl1	−15.88 (17)	Cu1—N1—C15—C14	−177.01 (19)
O1—Cu1—O6—Cl1	75.56 (16)	C13—C14—C15—N1	0.7 (4)
N1—Cu1—O6—Cl1	165.97 (17)	C16—C14—C15—N1	176.6 (2)
N3—Cu1—O6—Cl1	−101.95 (16)	C17—N2—C16—C14	−68.1 (3)
O1—Cu1—N1—C11	45.5 (2)	C18—N2—C16—C14	169.3 (2)
N3—Cu1—N1—C11	−138.1 (2)	C15—C14—C16—N2	123.4 (3)
O6—Cu1—N1—C11	−44.05 (19)	C13—C14—C16—N2	−60.9 (3)
O1—Cu1—N1—C15	−137.0 (2)	C18—N2—C17—C18^i^	−58.4 (3)
N3—Cu1—N1—C15	39.4 (2)	C16—N2—C17—C18^i^	178.3 (2)
O6—Cu1—N1—C15	133.5 (2)	C17—N2—C18—C17^i^	58.2 (3)
O3—Cu1—N3—C21	−103.2 (2)	C16—N2—C18—C17^i^	−177.4 (2)
N1—Cu1—N3—C21	81.5 (2)	C25—N3—C21—C22	−1.7 (4)
O6—Cu1—N3—C21	−4.0 (2)	Cu1—N3—C21—C22	174.1 (2)
O3—Cu1—N3—C25	72.62 (18)	N3—C21—C22—C23	1.3 (4)
N1—Cu1—N3—C25	−102.68 (19)	C21—C22—C23—C24	0.6 (4)
O6—Cu1—N3—C25	171.78 (18)	C22—C23—C24—C25	−2.1 (4)
Cu1—O1—C1—O2	173.27 (17)	C22—C23—C24—C26	−178.9 (3)
Cu1—O1—C1—C2	−9.9 (3)	C21—N3—C25—C24	0.0 (4)
O2—C1—C2—C5	−7.0 (3)	Cu1—N3—C25—C24	−175.96 (19)
O1—C1—C2—C5	176.1 (2)	C23—C24—C25—N3	1.9 (4)
O2—C1—C2—C3	−129.6 (2)	C26—C24—C25—N3	178.8 (2)
O1—C1—C2—C3	53.5 (3)	C28—N4—C26—C24	−56.9 (3)
O2—C1—C2—C4	113.4 (3)	C27—N4—C26—C24	−179.2 (2)
O1—C1—C2—C4	−63.5 (3)	C25—C24—C26—N4	139.4 (2)
Cu1—O3—C3—O4	−168.75 (18)	C23—C24—C26—N4	−43.9 (4)
Cu1—O3—C3—C2	13.4 (3)	C28—N4—C27—C28^ii^	59.3 (3)
C5—C2—C3—O4	3.3 (3)	C26—N4—C27—C28^ii^	−175.1 (2)
C1—C2—C3—O4	126.8 (2)	C27—N4—C28—C27^ii^	−57.8 (3)
C4—C2—C3—O4	−116.5 (3)	C26—N4—C28—C27^ii^	179.7 (2)
C5—C2—C3—O3	−178.8 (2)		

Symmetry codes: (i) −*x*+2, −*y*+2, −*z*; (ii) −*x*+1, −*y*+2, −*z*+1.

### Hydrogen-bond geometry (Å, °)

**Table:**

*D*—H···*A*	*D*—H	H···*A*	*D*···*A*	*D*—H···*A*
O1W—H1WA···O12	0.894 (18)	1.98 (2)	2.838 (4)	160 (4)
O1W—H1WB···O8^iii^	0.875 (18)	2.35 (3)	3.053 (4)	137 (3)
O1W—H1WB···O2^iv^	0.875 (18)	2.46 (3)	3.120 (3)	133 (3)
O2W—H2WA···O7	0.929 (19)	2.14 (2)	3.044 (4)	164 (4)
O2W—H2WB···O1W^v^	0.941 (19)	1.95 (3)	2.807 (5)	150 (5)
N2—H2N···O2^vi^	0.887 (17)	1.804 (18)	2.673 (3)	166 (3)
N4—H4N···O4^vii^	0.923 (17)	1.727 (17)	2.647 (3)	175 (3)

Symmetry codes: (iii) −*x*, −*y*, −*z*+1; (iv) −*x*+1, −*y*, −*z*+1; (v) *x*+1, *y*, *z*−1; (vi) −*x*+2, −*y*+1, −*z*; (vii) −*x*+1, −*y*+1, −*z*+1.

## References

[bb1] Bruker (2007). *SMART* and *SAINT* Bruker AXS Inc., Madison, Wisconsin, USA.

[bb2] Johnston, L. L., Martin, D. P. & LaDuca, R. L. (2008). *Inorg. Chim. Acta*, **361**, 2887–2894.

[bb3] Palmer, D. (2007). *Crystal Maker* Crystal Maker, Bicester, Oxfordshire, England.

[bb4] Pocic, D., Planeix, J.-M., Kyritsakas, N., Jouaiti, A., Abdelaziz, H. & Wais, M. (2005). *CrystEngComm*, **7**, 624–628.

[bb5] Sheldrick, G. M. (1996). *SADABS* University of Göttingen, Germany.

[bb6] Sheldrick, G. M. (2008). *Acta Cryst.* A**64**, 112–122. 10.1107/S0108767307043930 18156677

